# An iliopsoas abscess caused by *Parvimonas micra*: a case report

**DOI:** 10.1186/s13256-019-2004-0

**Published:** 2019-03-01

**Authors:** Toyomitsu Sawai, Satoru Koga, Shotaro Ide, Sumako Yoshioka, Nobuko Matsuo, Hiroshi Mukae

**Affiliations:** 1 Department of Respiratory Medicine, Nagasaki Harbor Medical Center, 6-39 Shinchi-machi, Nagasaki, 850-8555 Japan; 2 Second Department of Internal Medicine, Nagasaki University Hospital, 1-7-1 Sakamoto-machi, Nagasaki, Japan

**Keywords:** Primary iliopsoas abscess, *Parvimonas micra*, CT-guided percutaneous drainage, Ampicillin/sulbactam

## Abstract

**Background:**

*Parvimonas micra*, a Gram-positive anaerobic coccus, is a rare pathogen for psoas abscess. We describe a case of a patient with iliopsoas abscess caused by *P. micra*.

**Case presentation:**

An 81-year-old Asian man presented to our department with complaints of fever since the preceding day. Abdominal computed tomography revealed the presence of a low-density mass in the right iliopsoas muscle indicative of a psoas abscess. Computed tomography-guided percutaneous drainage of the psoas abscess was performed. Results of organism cultures of the abscess and blood were positive for *P. micra*. However, our patient had no known primary focus of infection. On the basis of these findings, a primary psoas abscess caused by *P. micra* was diagnosed, and treatment with ampicillin/sulbactam 1.5 g, administered intravenously every 8 h, was initiated. By day 7, the patient’s white blood cell count normalized. By day 20, his C-reactive protein level was decreased to 0.35 mg/dl.

**Conclusion:**

Iliopsoas abscesses caused by anaerobic bacteria are relatively rare, and iliopsoas abscesses caused by *P. micra* are especially rare. Our patient’s case revealed that *P. micra* can cause iliopsoas abscess. Therefore, clinicians should be aware of the possibility that *P. micra* may cause iliopsoas abscess.

## Introduction

*Parvimonas micra*, formerly known as *Peptostreptococcus micros* and *Micromonas micra*, is a fastidious, anaerobic, Gram-positive coccus that is normally found in the human dental and gastrointestinal flora [1]. *P. micra* is related to polymicrobial infections, especially in the oral cavity. It has also been implicated in more invasive infections, such as vertebral osteomyelitis, spondylodiscitis, arthritis, endocarditis, and pleuritis [2–5]. Although a variety of infections associated with *P. micra* have been reported, psoas abscesses caused by this organism have been described only rarely. We report a case of a patient with iliopsoas abscess caused by *P. micra.*

## Case presentation

**Fig. 1:**
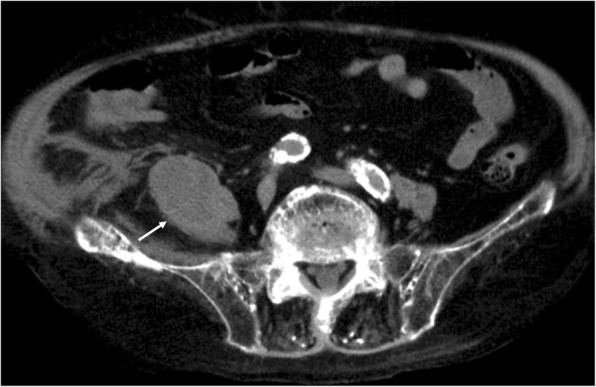
Abdominal computed tomography on admission showed a low-density mass in the right iliopsoas muscle (*white arrow*)

An 81-year-old Asian man presented to our department complaining of fever since the preceding day. The patient had been under treatment for the previous 3 years for chronic heart failure and chronic renal failure. He did not have a history of malignancy, diabetes mellitus, cytotoxic therapy, or corticosteroid use, and no foreign bodies had been implanted. The patient’s family history was unremarkable. Physical examination revealed a heart rate of 101 beats/min, blood pressure of 87/48 mmHg, respiratory rate of 20 breaths/min, temperature of 37.0 °C, and oxygen saturation of 87% on room air. He had no caries or periodontitis. Results of respiratory, cardiac, and abdominal examinations were unremarkable. Limb examination demonstrated mild edema of both legs. Abdominal computed tomography (CT) showed a low-density mass in the right iliopsoas muscle indicative of an iliopsoas abscess (Fig. 1). The patient’s white blood cell count, C-reactive protein (CRP), and procalcitonin levels were 19,400/μl, 13.35 mg/dl, and 3.950 ng/ml, respectively. Serum blood urea nitrogen and creatinine were elevated at 77.2 mg/dl and 3.69 mg/dl, respectively. A CT-guided percutaneous drainage of the psoas abscess was performed, and an indwelling catheter was placed. Gram staining of the drained fluid revealed many neutrophils and Gram-positive streptococci. On the basis of these findings, a presumptive diagnosis of iliopsoas abscess caused by *Streptococcus* species was made, and treatment with ampicillin/sulbactam (ABPC/SBT) 1.5 g, administered intravenously every 8 h, was initiated. Results of organism cultures of the abscess and blood were positive, and *P. micra* was identified by using the API ZYM system (Sysmex-bioMérieux Co. Ltd., Tokyo, Japan), with the organism exhibiting susceptibility to penicillin G, ampicillin, clindamycin, and meropenem. By day 7, the patient’s white blood cell count normalized. By day 20, his CRP level was decreased to 0.35 mg/dl. Therefore, the pigtail catheter was removed. The patient died of peritonitis due to colon diverticulum perforation after 5 weeks of treatment. An autopsy revealed no right iliopsoas abscess at the time of death.

## Discussion

Iliopsoas abscess is a rare life-threatening infection with a varied symptomology and etiology. The diagnosis and effective management are frequently delayed because of the nonspecific nature of clinical symptoms. Primary and secondary iliopsoas abscesses are most frequently caused by *Staphylococcus aureus* and *Escherichia coli*, respectively [6, 7]. A review of the literature showed that there have been very few cases of iliopsoas abscesses caused by *P. micra* [7, 8]. Our patient’s case had no known primary focus of infection. Given that the iliopsoas abscess in our patient apparently arose as a result of hematogenous dissemination from an unknown distant focus of infection, a diagnosis of primary iliopsoas abscess was made. Navarro Lopez *et al.* reported that *Peptostreptococcus* species were isolated from 5 of 72 (6.9%) patients with secondary iliopsoas abscess, whereas this organism was isolated from 1 of 21 (4.8%) patients with primary iliopsoas abscess [8]. Wada *et al.* reported a case of primary *P. micra* iliopsoas abscess due to hematogenous dissemination from a central venous catheter [9]. However, Wada *et al*. did not collect samples from the abscess, and *P. micra* iliopsoas abscess was diagnosed solely on the basis of isolation from the patient’s blood culture. In a review of 31 case reports with infections caused by *P. micra*, Cobo *et al*. reported that the infection site was the vertebral spine in 14 (45.1%) cases; joints and heart valves in 5 (16.1%) cases each, pleura in 3 (9.6%) cases; and in brain, lung and head and neck, chest wall, or meninges in 1 (3.2%) case each. A case of iliopsoas abscess had not previously been reported [5]; accordingly, our patient’s case appears to be a definitive case of a primary iliopsoas abscess caused by *P. micra*.

Iliopsoas abscess remains a relatively uncommon clinical entity. Since Mynter’s original description of pyogenic iliopsoas abscess in 1881 [10], more than 500 cases have been reported in the world literature [8, 11, 12]. The classic triad of back pain, limp, and fever may be present, but subsequent case studies have suggested that this triad may be detected in only 30% of cases [13]. Indeed, in our patient, the only clinical symptom was fever. Many patients with psoas abscess will present with nonspecific features such as malaise and low-grade pyrexia, meaning that there frequently may be delays in diagnosis. Accordingly, CT has become the standard means of diagnosing psoas abscess, with a sensitivity of over 90% and specificity of over 80% [14]. Although magnetic resonance imaging (MRI) has been shown to be more sensitive than CT in detecting intra-abdominal abscess [15], MRI is complex, and radiologists are not freely able to report on MRI. An additional advantage is that CT guidance allows safe percutaneous needle placement and the collection of samples from the psoas abscess. Our patient was diagnosed by using abdominal CT, and CT-guided percutaneous drainage was performed.

Blood cultures and direct abscess aspirate are the most definitive diagnostic tools. However, a definitive microbiological diagnosis for psoas abscess has been reported in only 75% of cases. Specifically, the rates of diagnosis by infected fluid and blood culture have been reported to be 74.3% and 31.5%, respectively [8]. *S. aureus* and *E. coli* are the most common organisms detected in primary and secondary iliopsoas abscess, respectively. Iliopsoas abscesses caused by anaerobic bacteria are relatively rare, and iliopsoas abscesses caused by *P. micra* appear to be especially rare. The anaerobe that most often causes anaerobic iliopsoas abscess is *Bacteroides* species [8]. However, *P. micra* might be an underreported causative pathogen in iliopsoas abscesses, because the organism is difficult to culture and identify. Cobo *et al.* reported that *P. micra* infections were diagnosed on the basis of infected fluids in 38.7% of cases, blood cultures in 29%, and molecular techniques in 22.5% [5]. In recent times, as new methods (e.g., 16S ribosomal RNA sequencing [3], matrix-assisted laser desorption ionization time-of-flight mass spectrometry [4], and melting temperature mapping [16]) have become more available in routine diagnostic laboratories, *P. micra* has been increasingly detected in various invasive human infections.

Although the first-line treatment in the literature is broad-spectrum antibiotic that will cover *S. aureus* and also any other possible primary source for the iliopsoas abscess, some authors have suggested that targeted antibiotics may be sufficient to treat abscesses up to 60 mm in width [17]. Additionally, it has since been established that image-guided percutaneous drainage is very effective and safe [18]. Post-drainage antibiotic therapy should be tailored to the organism isolated. The treatment for iliopsoas abscess caused by *P. micra* includes antimicrobial therapy with or without drainage. However, a standard antibiotic therapy for *P. micra* infections has not yet been established. *P. micra* is usually susceptible to antibiotics, including penicillin, imipenem, clindamycin, and metronidazole, although metronidazole-resistant strains of *P. micra* have been reported [19–21]. In general, metronidazole should not be administered as empiric therapy until susceptibility testing results are available. Accordingly, we selected intravenous ABPC/SBT antibiotic therapy with drainage of the abscess, a regimen that successfully treated the abscess in our patient. However, the optimal duration of therapy for iliopsoas abscess is unknown.

## Conclusions

Iliopsoas abscesses caused by anaerobic bacteria are relatively rare, and iliopsoas abscesses caused by *P. micra* appear to be especially rare. Our patient’s case revealed that *P. micra* can cause primary iliopsoas abscess. Therefore, clinicians should be aware of the possibility that *P. micra* may cause primary iliopsoas abscess.

## Abbreviations

ABPC/SBT: Ampicillin/sulbactam
CRP: C-reactive protein
CT: Computed tomography
MRI: Magnetic resonance imaging
